# Effect of Long-Term High Temperature Oxidation on the Coking Behavior of Ni-Cr Superalloys

**DOI:** 10.3390/ma11101899

**Published:** 2018-10-04

**Authors:** Stamatis A. Sarris, Manjunath Patil, Kim Verbeken, Marie-Françoise Reyniers, Kevin M. Van Geem

**Affiliations:** Department of Materials, Textiles and Chemical Engineering, University of Gent, Technologiepark 914, 9052 Zwijnaarde, Belgium; Stamatis.Sarris@ugent.be (S.A.S.); manjunath.patil@ugent.be (M.P.); MarieFrancoise.Reyniers@ugent.be (M.-F.R.); Kevin.VanGeem@ugent.be (K.M.V.G.)

**Keywords:** Ni-Cr alloys, steam cracking, metallurgic aging, thermal cracking, ethane, coke formation, jet stirred reactor

## Abstract

The service time of an industrial cracker is strongly dependent on the long-term coking behavior and microstructure stability of the reactor coil alloy. Super alloys are known to withstand temperatures up to even 1400 K. In this work, several commercially available alloys have been first exposed to a long term oxidation at 1423 K for 500 h, so-called metallurgic aging. Subsequently, their coking behavior was evaluated in situ in a thermogravimetric setup under ethane steam cracking conditions (T_gasphase_ = 1173 K, P_tot_ = 0.1 MPa, X_C2H6_ = 70%, continuous addition of 41 ppmw S/HC of DMDS, dilution δ = 0.33 kg_H2O_/kg_HC_) and compared with their unaged coking behavior. The tested samples were also examined using scanning electron microscopy and energy diffractive X-ray for surface and cross-section analysis. The alloys characterized by increased Cr-Ni content or the addition of Al showed improved stability against bulk oxidation and anti-coking behavior after application of metallurgic aging due to the formation of more stable oxides on the top surface.

## 1. Introduction

Surface technologies to reduce carbon deposition on the inner wall of industrial steam crackers are at the foreground of research because of their potential for improving the process economics. Due to coke deposition, the reactor cross-sectional area is reduced, leading to excessive pressure drop over its length. The latter results in a change in ethylene selectivity [1,2], while the coke layer decreases heat transfer from the furnace to the reactors. To keep the cracking severity stable over time and to compensate for the additional conductive heat resistance, the fuel flow rate to the furnace burners has to be increased, which makes that the reactor tube metal temperature rises over time. When the coke or the temperature of the coil is approaching the maximum allowable value, a decoking procedure with a steam/air mixture is necessary [3,4,5]. This cyclic cracking-decoking operation affects the annual production capacity if the plant is furnace limited, resulting in higher operating costs.

Steam cracking reactors are typically made out of Fe-Ni-Cr alloys that exhibit excellent mechanical strength, resistance to thermal creep deformation, surface stability and resistance to corrosion, oxidation and coke formation [6,7]. Many authors have investigated the relation between the elemental composition and microstructure of several alloys and coatings under oxidizing or carburizing environments [8,9,10,11,12,13,14]. To date, several anti-coking technologies have been introduced and tested in the field of steam cracking, such as three-dimensional (3D) reactor technologies [15,16,17,18,19,20] including application of coatings [21,22,23,24,25,26,27,28,29,30,31,32,33,34], feed additives [35,36,37,38,39,40,41] and surface technologies [23,31,42] or combination of them. In 3D reactor technologies, the reactor tube geometry is altered from the conventional straight tube to a more elaborated geometry enhancing heat transfer or mass transfer phenomena. Different finned [15,16,20], ribbed [18], partially ribbed [19] and swirl flow tubes [15,16,17,18,19,20] have been investigated. The additives category is one of the most famous to reduce coke deposition. Sulfur-containing compounds are the most commonly applied group of additives [35,37,38,39,40,41,43]. The role of sulfur additives on diminishing carbon monoxide formation is well established, but their effect on coke formation is debated. Besides sulfur-containing additives, components with phosphorus [44,45] and silicon [35] have also been investigated. Often aluminum and manganese are added to enhance the coking resistance of an alloy aiming at the formation of protective oxide layers [6]. Alternatively, a thin layer of a coating can be deposited on the reactor base alloy surface. Distinction can be made between barrier coatings that passivate the inner wall [24,25,28,29,30] and catalytic coatings [23,26,27,31,32,33,34] that convert coke to carbon oxides. A barrier coating pacifies the base alloy by covering the catalytically active sites, eliminating catalytic coke formation. However, the non-catalytic coke formation through a free-radical mechanism—often termed pyrolytic coke- is not prevented. In contrast, catalytic coatings eliminate the base alloy catalytic coke formation by covering the original active sites and provide catalytic sites for converting radically formed coke to carbon oxides and hydrogen by reaction with steam via gasification reactions. Hence, a positive catalytic activity is added to the elimination of the negative catalytic activity of the base alloy.

The focus of most of the technologies above mentioned is on the initial—fresh–coking behavior of the alloy, but not on its long-term behavior. Further scientific studies evaluated the coking performance of different alloys and coatings under industrially relevant conditions [23,44,46,47,48,49,50]. However, the coking behavior after aging is not experimentally correlated with the microstructure of the samples in any of the studies. In the literature, typically only the effect on the microstructure and on the mechanical properties of the alloys is evaluated in long-term oxidation studies. Nevertheless, the coking behavior of an alloy when the temperature is close at its metallurgic limits is obviously of great importance and therefore it is studied in this work. The experimental data are compared both with non-aged and cyclically aged materials. SEM and EDX observations on both the surface and the cross sectional oxidation of the alloys as well as their microstructure allows for a better understanding of the studied phenomena. 

## 2. Experimental Section

### 2.1. Electro Balance Unit

The coking experiments were performed at the jet stirred reactor set-up of the Laboratory for Chemical Technology (LCT) of Ghent University. The Jet-stirred reactor (JSR) is designed in order to carry out thermogravimetric analyses on different reactor materials. Coke formed during the experiments is measured as a function of time under a broad range of conditions covering most of the industrial cases. The unit is capable of evaluating the effect of different pretreatment conditions (e.g., application of additives, temperature and duration of the pretreatment) as well as varying cracking conditions (e.g., the use of feed additives, steam dilution and cracking temperature) on coke formation, product distribution together with surface composition and morphology. 

**Figure 1:**
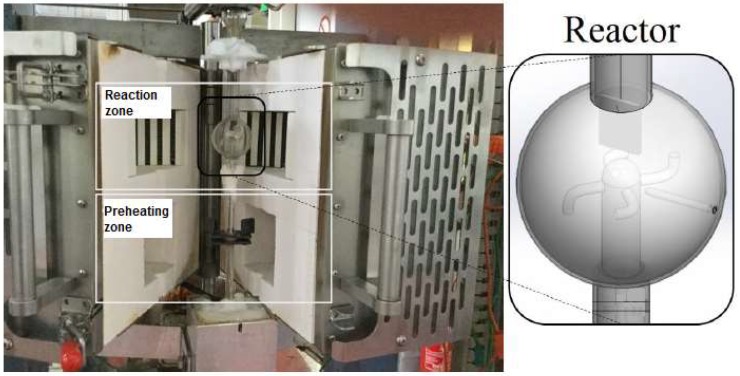
Illustration of the JSR reactor.

During an experiment the coke deposited on the metal sample, see Figure 1, and is accurately monitored online with resolution up to 0.1 μg per second. The weight signal is off-line processed afterwards in order to obtain coking rates. The reactor effluent is online analyzed via gas chromatography (GC) to evaluate the stability and reproducibility of the cracking conditions. The surface morphology and chemical composition of the coupons are typically analyzed off-line by means of SEM and EDX. The combination of the mentioned unit together with the experimental procedure gives valuable information on the coke formation phenomenon occurring during steam cracking of hydrocarbons and allows understanding the role of the studied material and conditions.

**Figure 2:**
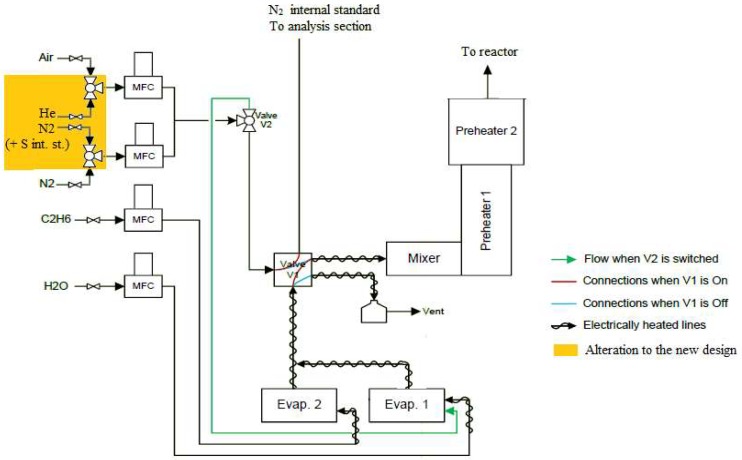
Diagram of the JSR feed and preheating section for the study of coke deposition on different reactor materials and pretreatments during ethane steam cracking.

The experimental unit is described in detail in earlier work [5,41,51]; however, for practical considerations a short description with the adaptation of some minor parts is included. Figure 2 gives a simplified diagram of the feed section of the unit.

In the feed section, the hydrocarbon feedstock and water (or water with DMDS) are fed, evaporated at 523 K, mixed at 543 K and preheated at 903 K—which is the highest temperature that cracking does not occur—before sent to the reaction section, where steam cracking occurs leading to coke formation on the sample. The reactor effluent is quenched using an oil cooler set at 473 K to prevent liquefaction of the products while fraction of the effluent is withdrawn for online carbon oxides, *C*_4_^−^ and *C*_5+_ GC analysis. The rest is sent directly to the vent.

### 2.2. Metallurgic Aging

In this work, an additional oven was used to off-line pretreat the coupons that subsequently were tested in the JSR. In this oven, high temperature oxidation and/or nitration can be performed. The oven is capable of withstanding temperatures up to 1600 K for more than 500 h.

### 2.3. Cyclic Aging

**Figure 3:**
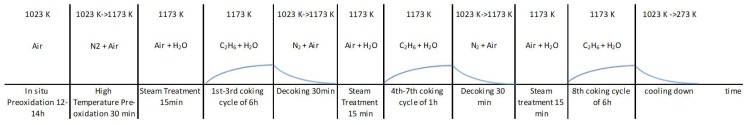
Timeline of a cyclic aging experiments.

Typically, the experiments conducted on the JSR unit consist of in total 8 cracking cycles—3 cycles of 6 h coking followed by decoking, followed by four short cycles of 1 h cracking and then decoking and a last cycle of 6 h—to evaluate the effect of cyclic cracking and decoking on each applied set of conditions. This procedure is called cyclic aging. An overview of the sequence kept in the aging experiments is given in Figure 3. In Table 1 and Table 2 detailed process conditions of the cyclic and metallurgic aging procedures are summarized.

### 2.4. Samples: Materials and Preparation

**Figure 4:**
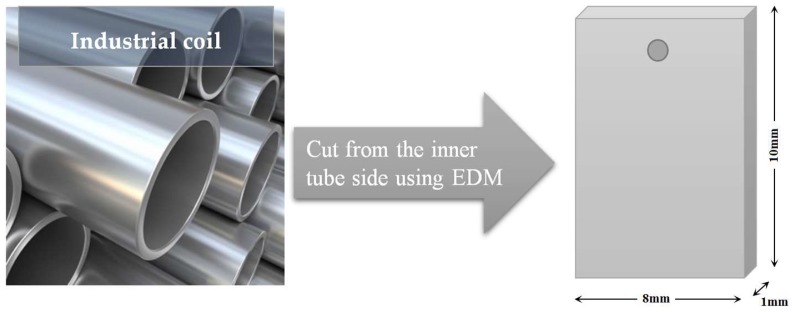
Illustration of JSR coupons sampling.

The metal coupons were made out 25/35 Cr/Ni, 35/45 Cr/Ni and 40/48 Cr/Ni alloys. Additionally, an Al-containing alloy is evaluated. These coupons are exposed to cyclic and metallurgic aging experiments. The coupons on which the coke will be deposited are cut as close as possible to the internal surface of industrial tubes using wire-cut electrical discharge machining (EDM) with an accuracy of 1 μ in dimensions 10 × 8 × 1 mm³ (total surface area S = 1.96 × 10^−4^ m^2^). An illustration of the JSR coupon is depicted in Figure 4.

### 2.5. Coking Rate Determination

During the cracking experiment various data is logged, such as temperatures, mass flow rates and the evolution of the mass of the coupon and thus the deposited coke. Due to the movement of the coupon during cracking the raw balance output contains high frequency noise, which is removed by a low-pass filter in Matlab, resulting in a filtered weight curve. Since the raw balance output in the first 15 min of cracking is unstable due to the transient start-of-run effects the coking data is processed after these first 15 min. The filtering step also serves to withhold large disturbances from the raw coking data and to reduce the amount of data points. Next, the filtered weight curve is regressed by minimizing the total sum of squares to the following equation with parameters *A*, *B*, *C* and *D*:

(1) $$W=At+B\left(1-\frac{1}{2}\left(e^{-Ct}+e^{-Dt}\right)\right)$$

Equation (1) is called the fitted weight curve, with *W* the amount of coke (10^−6^ kg) on the surface at time *t* (s). Via the fitted weight curve, data points for the first 15 min are calculated. The corresponding coking rate curve can be obtained by differentiating Equation (1):

(2) $$r_{c,\;coupon}=\frac{dW}{dt}=A+\frac{B}{2}\left(Ce^{-Ct}+De^{-Dt}\right)\;\left(\frac{10^{-6}kg}{s}\right)$$

The reported coking rate is then easily obtained by converting to the desired units:

(3) $$r_{c}=\frac{1000}{3600S_{coupon}}r_{c,coupon}\;\left(\frac{10^{-6}kg}{sm^{2}}\right)$$

With $S_{coupon}$ the surface area of the coupon in m^2^. The coking rate can also be calculated as a discrete derivative from the fitted weight curve as:

(4) $$r_{c}=\frac{1000}{3600S_{coupon}}\left(\frac{W_{t}{}_{i}-W_{t}{}_{i-1}}{t_{i}-t_{i-1}}\right)\;\left(\frac{10^{-6}kg}{sm^{2}}\right)$$

From 15–60 min, the calculated coking rate is considered to be representative of the catalytic behavior of the material, while the one calculated from 5th to 6th h is referring to the long-term behavior of the material, corresponding to pyrolytic coking occurring when the complete surface of the coupon is covered by coke. In this work, the former is referred as initial coking rate while the latter as asymptotic.

### 2.6. Surface Characterization

SEM and EDX (JEOL JSM-7600F FEG SEM, Tokyo, Japan) are used to obtain information regarding the surface morphology and elemental composition of the samples. Both top surface and cross section analyses of coked samples were performed in this work. The top surface analysis gives a qualitative idea of the coke growth together with elemental quantitative data of the surface, performed in 10 kV and 20 kV. On the other hand, the cross section mappings explain the uniformity of the oxides generated due to exposure to an oxidative or carburizing environment. In this work, during the cross sectional analyses also the microstructure of the metallurgical aged alloys is evaluated. In the EDX analysis, the detected oxygen and carbon are omitted in the results both not to block viable results for other elements such as Cr, Mn and Fe, but also because their quantitative determination is not possible via EDX.

## 3. Experimental Results

In Table 3 an overview of the calculated initial and asymptotic coking rates for all the different experiments is given. The results can be interpreted in many different ways; initially the effect of the different aging procedures on the coking rates, keeping as reference the 1 cc and as fixed variable the elemental composition and subsequently, the effect of the different elemental composition of the coupon on the coking rates for all different applied conditions. Additionally, the effect of the different applied conditions on the elemental composition can be discussed using the SEM and EDX top and cross sectional analyses. Therefore, in the next sections, a more detailed discussion of the results is given.

### 3.1. Coke Formation

**Figure 5:**
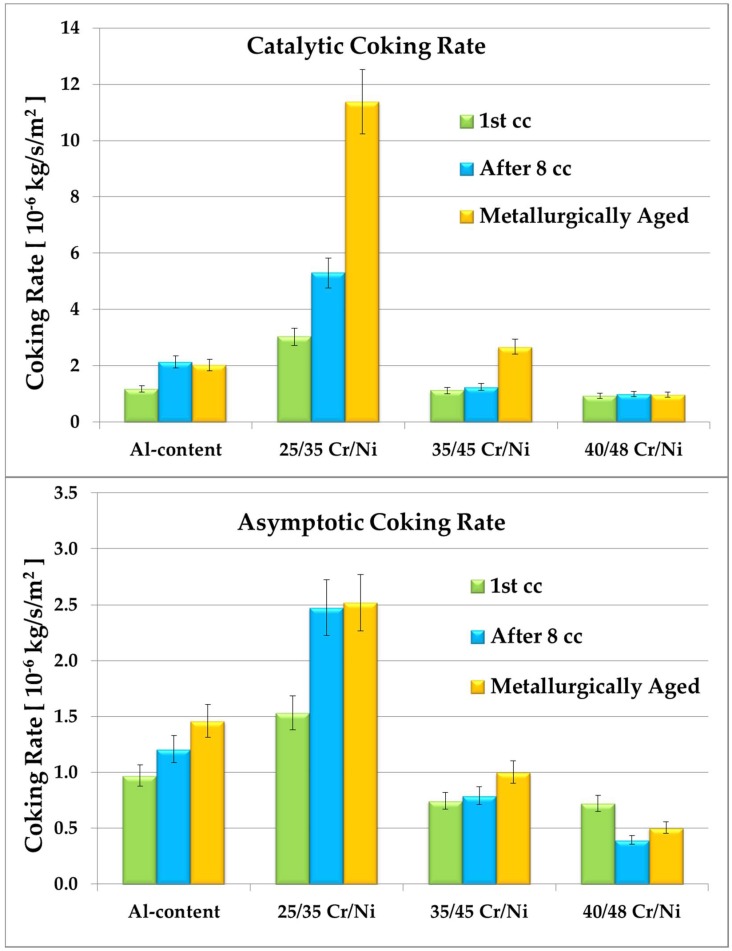
Coking rates for the 1st cracking cycle (green), the one after the cyclic aging (blue) and the one after metallurgic aging (orange).

In Figure 5 the coking behavior for the different alloys under the complete range of the applied conditions is shown. The worst anti-coking performance is observed for the 25/35 Cr/Ni alloy, showing in all conditions at least 3 times higher coking rates than the other materials. The best coking behavior is observed for the 40/48 Cr/Ni alloy that is at least 10% better than the competition. For the catalytic coking rate, during the 1st cc, the differences are only significant for the 25/35 Cr/Ni alloy, coking almost a factor 3 more than the rest. For the remaining alloys the differences are negligible for the catalytic coking rates; however the ascending ranking in order of anti-coking performance is the following:

25/35 Cr/Ni < Al-containing < 35/45 Cr/Ni < 40/48 Cr/Ni

Similar quantitative trends can be noted for the asymptotic coking rate, implying that the catalytic and pyrolytic coke formation are coupled phenomena. After application of cyclic aging even though the order of magnitude of the coking rates increases for all the alloys except for the 40/48 Cr/Ni one, the ranking of the materials remains the same. Here, the 25/35 Cr/Ni alloy cokes almost a factor 6 more than the best alloy, for both the catalytic and pyrolytic coking. Nevertheless, the differences remain similar among the other alloys. Application of cyclic aging leads to an increase of about double for the catalytic coking rates of the Al-containing and the 25/35 Cr/Ni alloy. For the asymptotic coking rate, a less pronounced increase of almost 30% is noted for these two alloys. For the remaining alloys—namely 35/45 and 40/48 Cr/Ni—the cyclic aging effect is negligible, implying their better anti-coking performance and stability during aging. The metallurgic aging, having as target to evaluate the materials after extreme oxidation conditions, similar to the end of run conditions of an industrial coil, is noted with orange in Figure 5. The anti-coking performance ranking of the alloys changes slightly when only considering the catalytic coking rate:

25/35 Cr/Ni < 35/45 Cr/Ni < Al-containing < 40/48 Cr/Ni

The Al-containing alloy cokes two times more than the high concentration of Cr/Ni alloys, while the 35/45 Cr/Ni alloy performs almost 15% worse than the former. The 25/35 Cr/Ni material remains the worst having almost a factor 10 higher coking rates. Unfortunately, the improved performance of the Al-containing alloy is not maintained during the pyrolytic coking stage. Here, the ranking is identical to the original one, with most of the alloys performing similarly to the cyclic aged ones, supporting the idea that the strongest factors influencing the radical pyrolytic coke formation are the cracking conditions, the applied pretreatment and the elemental composition, in agreement with previous observations [5].

### 3.2. Surface Analysis

**Figure 6:**
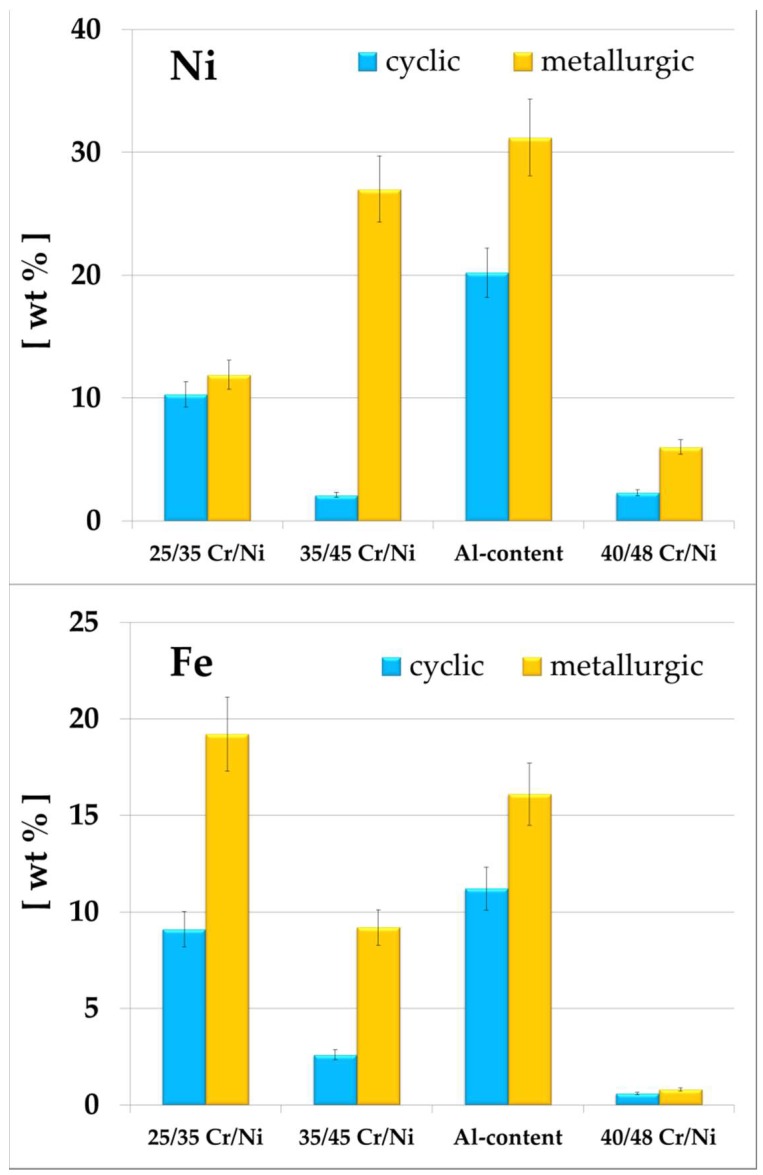
Ni content (**top**) and Fe content of the tested coked samples. Magnification: 50×, Accelerating voltage: 20 kV.

**Figure 7:**
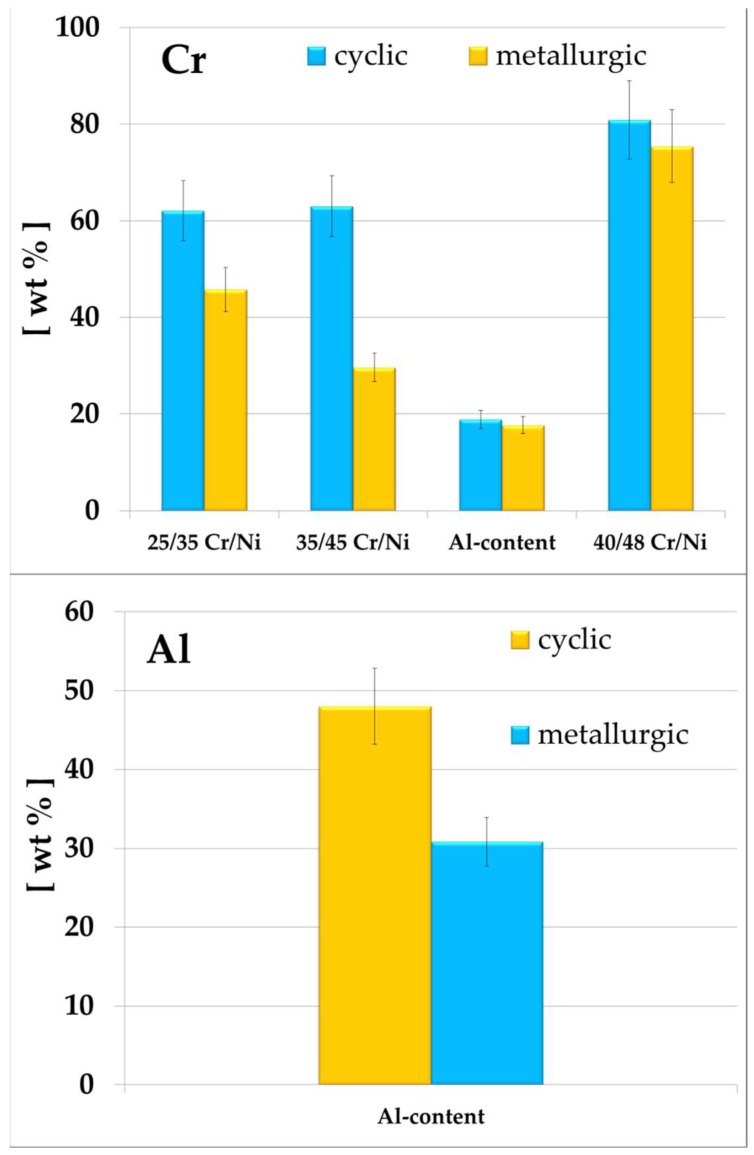
Cr content and Al-containing of the coked samples. Magnification: 50×, Accelerating voltage: 20 kV.

The coke formation observations are in line with the top surface elemental compositions measured by means of EDX, see Table 4. In Figure 6 Ni and Fe are chosen as the representative catalytic coking elements, while in Figure 7 Cr is picked for all alloys and Al is picked for the Al-containing, as representative for the passivating behavior [5,47]. The best anti-coking performance is measured for the 40/48 Cr/Ni alloy that has the lowest cumulative concentration of Fe and Ni on its surface, but also the highest amounts of Cr. The ranking of the tested alloys under the different conditions is fully aligned with the cumulative Fe and Ni content. It is clear that the existence of Cr in excess on the top surface of an alloy gives rise to the formation of Cr oxides having a good anti-coking performance. However, for the Al-containing no clear conclusions can be extracted. Certainly the absence of Fe and Ni in combination with the excess of elements responsible for the formation of oxides plays a significant role for the anti-coking performance of an alloy.

The ranking of the tested alloys under the different conditions is again fully aligned with the cumulative Fe and Ni content.

### 3.3. Cross Sectional Analysis

**Figure 8:**
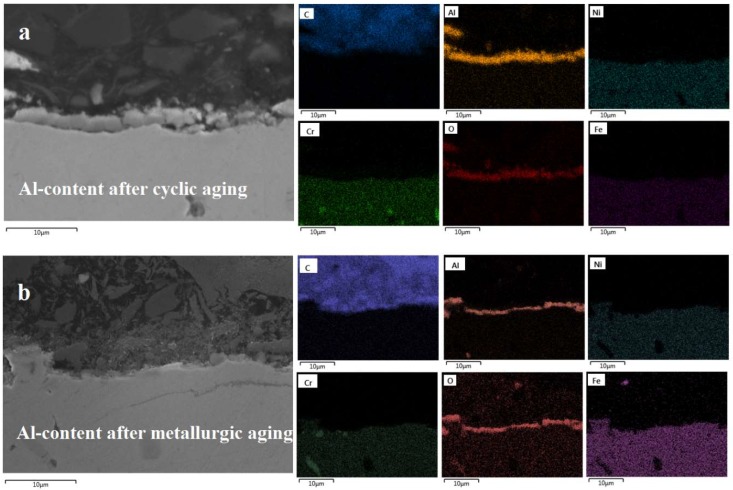
SEM-EDX images of an Al-containing alloy after cyclic aging (**a**) and after metallurgic aging (**b**). Magnification: 3000×, Accelerating Voltage: 15 kV.

With the exception of the top surface elemental composition of the tested alloys, the cross sectional elemental composition is evaluated. It is found that the homogeneity of the oxides formed on a surface is also responsible for its subsequent anti-coking performance [5]. From Figure 8, it is clear that the Al-containing alloy has a very nicely oxidized surface after application of cyclic aging, forming mainly an Al oxide of roughly 2 to 3 μm thickness. After application of the metallurgic aging the oxide remains quite homogeneous although in some points it is replaced or overlaps with Cr oxides. The thickness is also decreased by almost 1 μm. This is in agreement with the coking observations. Probably the overlaying of Cr and Al improves the initial catalytic coking behavior of the alloy after metallurgic aging; however it does not affect significantly the pyrolytic coking rate to have an impact on the ranking of the alloys.

**Figure 9:**
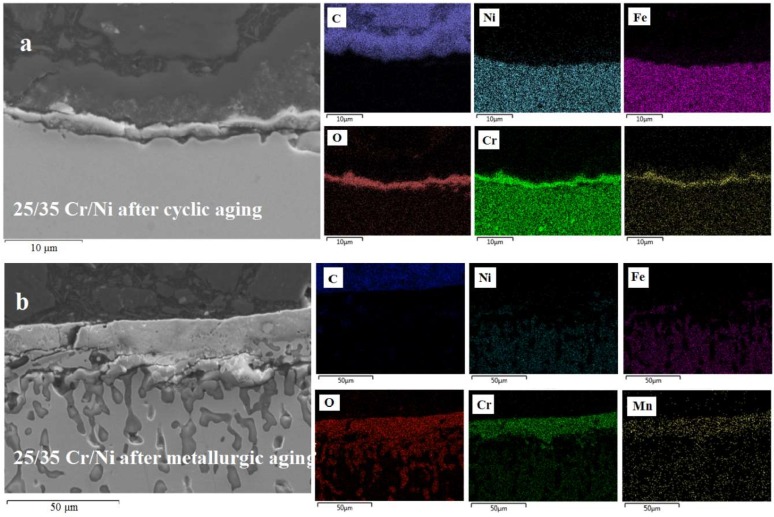
SEM-EDX images of a 25/35 Cr/Ni alloy after cyclic aging (**a**) and after metallurgic aging (**b**). Magnification: 3000× (**top**) and 1000× (**bottom**), Accelerating Voltage: 15 kV.

**Figure 10:**
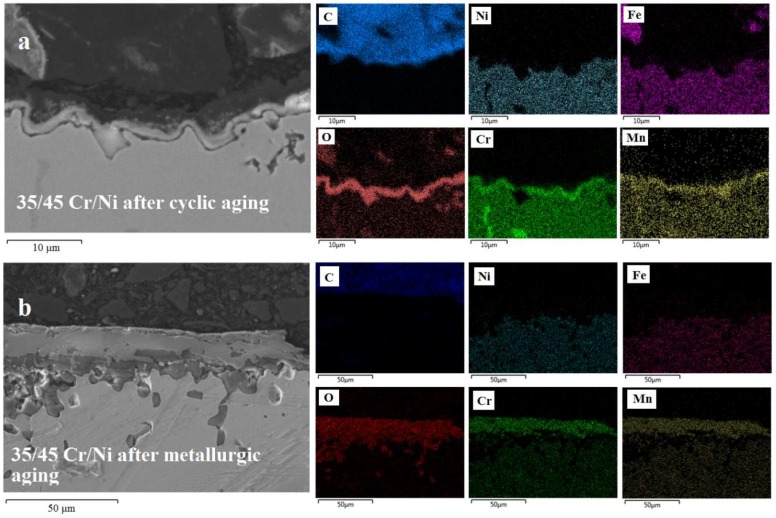
SEM-EDX images of a 35/45 Cr/Ni alloy after cyclic aging (**a**) and after metallurgic aging (**b**). Magnification: 3000× (**top**) and 1000× (**bottom**), Accelerating Voltage: 15 kV.

**Figure 11:**
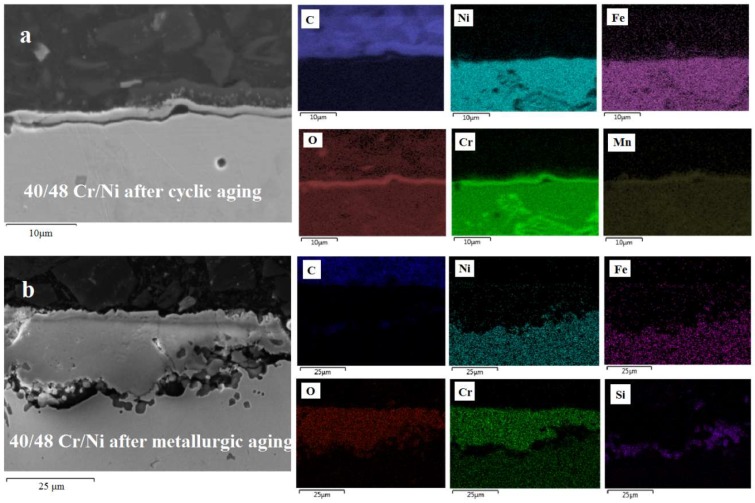
SEM-EDX images of a 40/48 Cr/Ni alloy after cyclic aging (**a**) and after metallurgic aging (**b**). Magnification: 3000× (**top**) and 1500× (**bottom**), Accelerating Voltage: 15 kV.

In Figure 9, Figure 10 and Figure 11, the superiority of the optimized pretreatment is vociferous. At the standard cyclic aging the alloys are forming a perfectly homogeneous Cr–Mn oxide layer of 2 to 3 μm thickness. Clearly the minor compositional differences imply that the Cr is less catalytic than Mn towards coke formation, while the increase in Ni is not enough to affect the coking performance of a material negatively if the latter is properly pretreated. A significant difference among the non Al and Al-containing alloys is the thickness of the oxides formed after the metallurgic aging. Evidently, the addition of Al in the composition of an alloy halts its oxidation at higher temperatures [48,52]. Oxygen cannot move towards the bulk as easily as in the case of the non Al ones, probably because Al_2_O_3_-α is formed that is rather stable at high temperature. This phenomenon seems to be more pronounced in the 25/35 Cr/Ni alloy and less in the 35/45 Cr/Ni one, while in the 40/48 is almost absent. Additionally, for the 25/35 Cr/Ni alloy, Ni and Fe overlay with the passivating oxides for metallurgical aged sample, obviously catalyzing coke formation in line with the measured coking rates.

The differences noted in the bulk of the alloys after exposure to the metallurgic aging process in the above mentioned cross sectional analyses led to the next section. This describes an attempt to identify possible microstructure patterns deeper in the bulk of the metallurgical aged alloys as a result of their exposure to temperatures close to their metallurgical limits.

### 3.4. Microstructure Analysis

**Figure 12:**
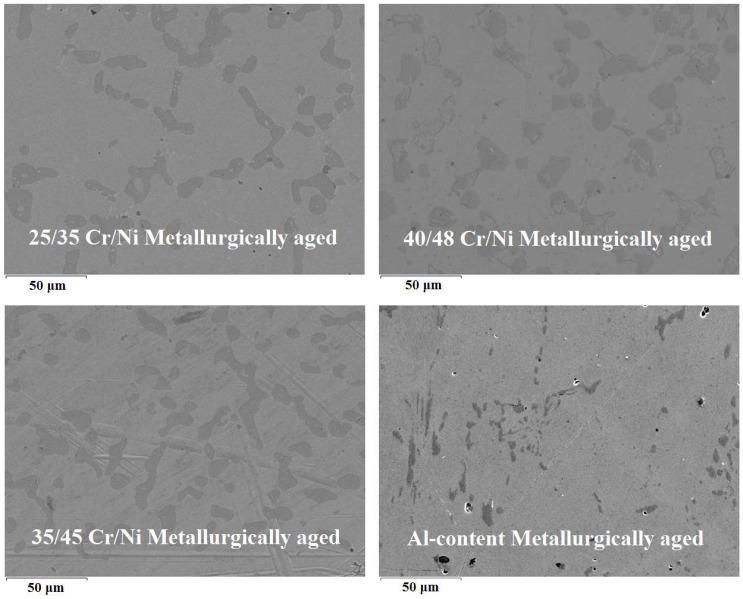
Microstructure SEM pictures after metallurgic aging. Magnification: 550×, Accelerating Voltage: 15 kV. Dark grey.

In Figure 12 the microstructure of the four alloys after exposure to the long-term oxidation treatment is shown where in comparison to the ones from Figure 8 to Figure 11 even at higher resolution no elaborated microstructure is observed. Please note that the black spots are spots with increased concentration in Si, Al and C, implying the existence of SiC or Al_4_C_3_. Additionally, also Cr-C rich zones, on the one hand, and Nb-C rich zones, on the other hand, are observed in the microstructure. The dark grey areas indicate a precipitation of Cr and C while the white ones corresponds to an increased concentration of Nb and C. It is known that metallurgic crack initiation after high temperature oxidation happens mainly in Chromium carbides rich zones [53,54]. Also, when the microstructure of an alloy is not significantly elaborated, the alloy is not significantly aged [55,56]. The most pronounced microstructure is noted for the Fe-Ni-Cr alloys, justifying that the microstructure of an alloy is independent of its coking behavior. The 25/35 and 40/48 Cr/Ni alloys have almost identical microstructure pattern, but totally different coking behavior after metallurgic aging, while the 35/45 is the most pronounced, even though its coking behavior is better than the former and worse than the latter. The Al-containing alloy shows no elaborated microstructure, but its coking behavior is still not the best. Therefore no clear conclusions can be made towards coke formation from the current microstructural analysis.

## 4. Conclusions

The coking behavior of four different alloys was evaluated in a thermogravimetric unit after their exposure to a long-term high temperature oxidation for 500 h more representative of the end of run conditions. The results indicated that for the same conditions the coking resistance is improved by increasing the content of Cr and Ni of a high temperature alloy. Cyclic aging had only a minor effect on the tested alloys except for the 25/35 Cr/Ni alloy. The anti-coking performance of the materials decreases after the metallurgic aging. For the best tested material, a negligible effect was noticed after application of the long-term oxidation. The catalytic and pyrolytic coking behavior of the materials is quite aligned, implying that the two phenomena are interconnected. In line with previous work and literature it can be concluded that the presence of Fe and Ni is detrimental for the anti-coking performance of an alloy. Most of the alloys indicated a rather homogeneous coverage of their surface by oxides—mainly of Cr nature for the non Al-containing alloys. For the Al-containing alloy, the thickness of the oxides covering typically the surface after cyclic aging is reduced by almost 1 μm, in contrast with the other alloys for which the oxides’ thickness was significantly increased. In line with the available literature, the addition of Al in the content of a high temperature alloy, forms Al oxides that protect the bulk from further oxidation. No microstructure was found in any alloy, which implies that the alloys all are quite resistant in terms of aging. However the non Al-containing alloys showed increased concentration of Carbides of Si and Nb in comparison with the Al-containing one. No correlation between the microstructure and the coking behavior of an alloy was found.

## Figures and Tables

**Table 1:** Schematic overview of the coking-decoking experimental procedure.

Process Step	Duration	Temperature (K)	Gas Feed Flow (10^−6^ kg s^−1^)			Water Flow (10^6^ kg s^−1^)
			N_2_	Ethane	Air	
**Preoxidation**	12–14 h	1023	-	-	9.575	-
**Decoking**	30–40 min	Heating up from 1023 to 1173	9.683	-	11.862	-
**Steam treatment**	15 min	1173	-	-	11.862	6.7
**1st cracking cycle**	6 h	1173	-	29.167	-	9.7
**Decoking**	30–40 min	Heating up from 1023 to 1173	9.683	-	11.862	-
**Steam treatment**	15 min	1173	-	-	11.862	6.7
**2nd cracking cycle**	6 h	1173	-	29.167	-	9.7
**Decoking**	30–40 min	Heating up from 1023 to 1173	9.683	-	11.862	-
**Steam treatment**	15 min	1173	-	-	11.862	6.7
**3rd cracking cycle**	6 h	1173	-	29.167	-	9.7
**Decoking**	30–40 min	Heating up from 1023 to 1173	9.683	-	11.862	-
**Steam treatment**	15 min	1173	-	-	11.862	6.7
**Cyclic aging**	Consists of 4 cycles of: ○ Heating up from 1023 to 1173 K ○ Cracking at 1173 K (1 h) ○ Decoking from 1023 to 1173 K ○ Steam treatment at 1173 K (15 min)					
**8th cracking cycle**	6 h	1173	-	29.167	-	9.7
**Decoking**	30–40 min	Heating up from 1023 to 1173	9.683	-	11.862	-
**Steam treatment**	15 min	1173	-	-	11.862	6.7
**Cooling down**	Not specified	To ambient temperature	-	-	-	-

**Table 2:** Procedure of metallurgic aging followed by a single coking-decoking cycle.

Process Step	Duration	Temperature (K)	Gas Feed Flow (10^−6^ kg s^−1^)			Water Flow (10^−6^ kg s^−1^)
			N_2_	Ethane	Air	
**Metallurgic aging**	500 h	1423	-	-	9.575	-
**High-temperature preoxidation**	30–40 min	Heating up from 1023 to 1173	9.683	-	11.862	-
**Steam treatment**	15 min	1173	-	-	11.862	6.7
**1st cracking cycle**	6 h	1148	-	29.167	-	9.7
**Decoking**	30–40 min	Heating up from 1023 to 1173	9.683	-	11.862	-
**Steam treatment**	15 min	1173	-	-	11.862	6.7
**Cooling down**	Not specified	To ambient temperature	-	-	-	-

**Table 3:** Summary of the main coking results.

Experiment		Cyclic Aging			
		Al-Containing	25/35 Cr/Ni	35/45 Cr/Ni	40/48 Cr/Ni
**Conditions**	**Ethane Flow [10^−6^ kg s^−1^]**	29.17			
	**Steam Flow [10^−6^ kg s^−1^]**	9.72			
	**CA DMDS amount (ppmw S/HC)**	41			
	**Temperature (K)**	1173			
	**Pressure**	1.02			
	**dillution**	0.33			
**Initial Coking Rate** **[10^−6^ kg/s/m^2^]**	**cc**				
	**1st cc**	1.18	3.03	1.13	0.94
	**After 8 cc**	2.14	5.29	1.24	0.99
	**metallurgical Aged**	2.03	11.38	2.67	0.97
**Asymptotic Coking Rate** **[10^−6^ kg/s/m^2^]**	**cc**				
	**1st cc**	0.97	1.53	0.74	0.72
	**After 8 cc**	1.21	2.47	0.79	0.40
	**Metallurgical Aged**	1.46	2.52	1.00	0.51

**Table 4:** Top surface elemental composition for the coked samples after cyclic and metallurgic aging. Magnification: 50×, Accelerating voltage: 10 and 20 kV.

Alloy		25/35 Cr/Ni		35/45 Cr/Ni		Al-Containing		40/48 Cr/Ni	
Elements	Acc. Volt.	Cyclic	Metallurgic	Cyclic	Metallurgic	Cyclic	Metallurgic	Cyclic	Metallurgic
Ni	10 kV	3.7	13.9	7.6	20.9	13.8	54.2	8	16.1
	20 kV	10.3	11.9	2.1	27	20.2	31.2	2.3	6
Fe	10 kV	1.6	19.1	2	8.2	7.5	22.8	1.3	0
	20 kV	9.1	19.2	2.6	9.2	11.2	16.1	0.6	0.8
Cr	10 kV	65.2	43.6	47.9	34.3	9.8	17.6	72.4	71.2
	20 kV	62.1	45.8	63	29.6	18.9	17.7	80.9	75.4
Si	10 kV	1.4	4.3	2.1	20.9	2.1	1.1	1.2	1.2
	20 kV	2.1	4.6	1.3	31.2	1.2	1.7	1	13.5
Mn	10 kV	27.8	17.7	40.4	1.1	-	-	16.9	11.6
	20 kV	15.5	17.1	30.7	0.7	-	1.9	14.8	4.1
Nb	10 kV	0.4	1.4	-	5.1	0.4	1.7	0.2	0
	20 kV	0.9	1.5	-	2.3	0.4	-	0.4	0.2
Al	10 kV	-	-	-	-	66.5	2.6	-	-
	20 kV	-	-	-	-	48	30.8	-	-

## Nomenclature

A	Atomic mass of element [g mol^−1^]
EDM	wire-cut electrical discharge machining
EDX	energy dispersive X-ray analysis
FID	flame ionization detector
F	Flow rate [10^−6^ kg s^−1^]
H	Penetration depth [µm]
cc	coking-decoking cycle number i
JSR	jet-stirred reactor
L	Nominal thickness [μm]
m_tj_	mass of coke at time j [kg]
Mc	amount of coke deposited on the sample [g]
P_tot_	reactor pressure [MPa]
P/E	ratio of propylene [wt. %] to ethylene [wt. %]
r_c_	coking rate [kg s^−1^ m^−2^]
r_c,initial_	initial catalytic coking rate [kg s^−1^ m^−2^]
r_c,asymptotic_	asymptotic coking rate [kg s^−1^ m^−2^]
r_x-y_	average coking rate in between the given time intervals [kg s^−1^ m^−2^]
RGA	refinery gas analyzer
S	surface area of the coupon [m^2^]
SEM	scanning electron microscopy
TCD	thermal conductivity detector
T	temperature [K]
V	Accelerating voltage [kV]
z	Atomic number
**Greek**	
δ	dilution [kg kg^−1^]
ρ	Density of the material [g cm^−3^]
τ	mean residence time [s]
